# Is intimate partner violence and obstetrics characteristics of pregnant women associated with preterm birth in Ethiopia? Umbrella review on preterm birth

**DOI:** 10.1186/s12978-023-01716-7

**Published:** 2023-11-17

**Authors:** Addis Eyeberu, Addisu Alemu, Adera Debella, Ibsa Mussa

**Affiliations:** 1 School of Nursing and Midwifery, College of Health and Medical Sciences, Haramaya University, Harar, Ethiopia; 2 School of Public Health, College of Health and Medical Sciences, Haramaya University, Harar, Ethiopia

**Keywords:** Preterm birth, Pregnant women, Umbrella review, Meta-analysis, Ethiopia

## Abstract

**Background:**

Preterm birth is a significant contributor to newborns morbidity and mortality. Despite the availability of highly effective and powerful interventions, the burden of preterm birth has not decreased. Given the relevance of the topic to clinical decision-making, strong conclusive and supporting evidence emanating from the umbrella review is required. To this end, this umbrella review study sought to determine the association between intimate partner violence and obstetrics characteristics of women with preterm birth in Ethiopia.

**Methods:**

Six systematic review and meta-analysis studies searched across multiple databases were included in this umbrella review. The quality of the included systematic review and meta-analysis studies was evaluated using the Assessment of Multiple Systematic Reviews (AMSTAR-2) checklist. STATA version 18 was used for the statistical analysis. A random-effects model was used to calculate the overall effect measurement.

**Results:**

A total of 114 observational studies in the six systematic review and meta-analysis studies involving 75,624 pregnant women were included in this comprehensive analysis. The preterm birth rate among mothers in Ethiopia was 11% (95% CI 10–13%; I2 = 98.08). Preterm birth was significantly associated with intimate partner violence (POR: 2.32; 95% CI 1.74–2.90), multiple pregnancies (POR: 3.36; 95% CI 2.41–4.32), pregnancy-induced hypertension (POR: 4.13; 95% CI 3.17–5.10), anemia (POR: 2.76; 95% CI 1.97–3.56), and premature rupture of pregnancy (POR: 5.1; 95% CI 3.45–6.75).

**Conclusions:**

More than one out of ten pregnant women experienced preterm birth in Ethiopia. Intimate partner violence is significantly associated with preterm birth. Furthermore, multiple pregnancies, pregnancy-induced hypertension, anemia, and premature rupture of the membrane were significant predictors of preterm birth. Therefore, policymakers should consider further instigations and implementations of policies and strategies closely related to reductions of intimate partner violence. It is also crucial to the early identification and treatment of high-risk pregnancies.

**Supplementary Information:**

The online version contains supplementary material available at 10.1186/s12978-023-01716-7.

## Introduction

Preterm delivery is a serious clinical issue that is linked to high newborn morbidity and mortality rates [1, 2]. Preterm births occur before 37 full weeks of pregnancy or less than 259 days following the start of a woman's last normal menstrual period (LNMP) [3]. Gestational age has a substantial impact on the burden of preterm birth. LNMP and early ultrasonography are the best methods for determining gestational age [3, 4]. Evidence showed that an estimated 13·4 million newborn babies were born preterm worldwide in 2020 [5]. The global annual rate of preterm birth reduction was estimated at—0·14% [5]. The two regions (Asia and sub-Saharan Africa) accounted for 65% of all preterm births globally in 2020 [5]. Africa has a wide range of preterm birth rates, ranging from 6.6% in Uganda to 16.6% in Tanzania [6]. The preterm birth rate in Ethiopia ranges from 8.76% [7] to 13.32% [8].

Numerous sociodemographic factors such as younger maternal age, rural residence, educational level, and marital status [9, 10]; nutrition-related factors such as malnutrition and anemia [11–13]; obstetric characteristics such as antenatal care visits, pregnancy-induced hypertension, antepartum hemorrhage, multiple pregnancy, and premature rupture of membranes [8, 10, 13]; behavior-related factors such as alcohol drinking and smoking [14, 15]; and environmental factors [16] have been shown to increase the risk of preterm birth. There is little evidence that violence is a risk factor for preterm birth [17]. Despite the abundance of identified risk factors, most preterm newborns have no obvious risk factor [3].

Preterm births have significant consequences for both neonates and mothers. Prematurity accounts for 35% of all neonatal mortality in the world [18]. Neonatal mortality is still high in Ethiopia which accounts for 30 neonatal deaths per 1000 live births [19]. Prematurity is a major cause of neonatal mortality in Ethiopia [20]. Aside from death, it can cause serious short- and long-term newborn concerns, such as breathing difficulties, feeding issues, brain damage, developmental delays, and vision and hearing impairment [21–23]. Furthermore, prematurity has a negative influence on the family and mothers, including social isolation, anxiety, exhaustion, and psychological anguish [24–26].

Ethiopia has introduced various measures to reduce the burden of preterm birth. Preconception care, early detection and treatment of women at risk of preterm birth, antenatal corticosteroid provision, prevention of unintended pregnancy, prevention of pregnant women being exposed to cigarette smoke, and prudent use of fertility treatments are some of the strategies that help reduce preterm birth [27, 28]. Additionally, the country made an unreserved effort to enhance neonatal intensive care (NICU)which significantly improved the survival of preterm newborns [1]. However, the standard of quality of care remains a significant barrier.

Despite these initiatives, the burden of premature birth remains unchanged. It is essential to review and reevaluate the current policies to improve the survival rate of premature newborns. Umbrella review is the strongest evidence that can help policymakers in developing novel measures that can reduce infant deaths associated with prematurity. To the authors' knowledge, no umbrella review done to investigated the relationship between intimate partner violence and obstetric characteristics of pregnant women and preterm birth in Ethiopia. Therefore, this umbrella review aimed to assess the overall state of preterm births in Ethiopia and explore the association between intimate partner violence and obstetric characteristics of pregnant women with preterm birth in Ethiopia.

## Methods

This umbrella review was established to provide decision-makers with a single, comprehensive source of information for use in formulating clinical recommendations, intervention strategies, and evaluating healthcare treatments following prior research [29]. Preferred Reporting Items for Systematic Reviews and Meta-Analyses (PRISMA) reporting guidelines were followed to report the study [30] (Additional file 1).

### Eligibility criteria

All suitable systematic reviews and meta-analyses (SRMA) studies on preterm delivery, intimate partner violence, and obstetrical characteristics of pregnant women who gave premature birth in Ethiopia were included. The pre-established inclusion criteria were as follows: the population was pregnant mothers and their newborns; the study area was only studies carried out in Ethiopia; the study design was all SRMAs studies; the publication condition was both published and unpublished research; and language was studies reported in English. Finally, all full-text articles published before August 13, 2023, were included. SRMA studies without well-defined study questions, search strategies, or article selection procedures were excluded. We also excluded narrative reviews, editorials, letters, abstracts, methodological studies, and literature reviews.

### Information sources

We used reliable databases like the Web of Sciences, CINAHL (EBOSCO), Science Direct, MEDLINE, EMBASE, SCOPUS, Google Scholar, and PubMed to conduct our electronic literature search. We also attempted to use direct Google searches to visit university repository websites.

### Search strategy

Two authors (AE and AD) searched for both published and unpublished SRMA studies using a combination of Boolean logic operators (AND, OR, NOT), Medical Subject Headings (MeSH), and keywords in the aforementioned databases: A thorough search was conducted using the PICOS questions. The PubMed search query was (("premature birth"[MeSH Terms] AND "pregnant women"[MeSH Terms]) OR "infant, newborn"[MeSH Terms]) AND ("systematic review"[Publication Type] OR "systematic reviews as topic"[MeSH Terms] OR "systematic review"[All Fields]) AND ("meta-analysis"[Publication Type] OR "meta-analysis as topic"[MeSH Terms] OR "meta-analysis"[All Fields]) AND "Ethiopia"[MeSH Terms].

### Selection process

All search results were exported to the EndNote X8 citation system, and duplicate publications were removed to find systematic reviews and meta-analyses that met the inclusion criteria. The whole text of the papers was evaluated after the publications were screened by title and abstract. Duplicate authors (AE and AD) individually examined each paper. A discussion with a third reviewer (AA) was used to resolve any disagreements among the authors regarding the admissibility of certain studies.

### Data extraction

Duplicate extraction was conducted for data from the included SRMA studies to assess study quality and evidence synthesis. Microsoft Excel 2016 was used to extract information about the publication period, number of studies included in each SRMA study, study design, sample size, risk of bias assessment technique, and predictors.

### Quality assessment of the systematic review and meta-analyzed studies

All pertinent systematic reviews and meta-analysis studies were evaluated for quality using the AMSTAR-2 (Assessment of Multiple Systematic Reviews) quality assessment method [31]. The tool has 16 components, including 9 noncritical and 7 critical domains. Critical domains include the protocol was registered before the review was started, the extent of the literature search, the rationale for excluding particular studies, the risk of bias from the studies included in the review, the suitability of meta-analysis methods, taking into account the risk of bias when interpreting the review's findings, and the assessment of the existence and likely consequences of publication bias [31]. In the tool, the possible answers are indicated as "Yes," "Partial Yes," "No," or "No Meta-analysis conducted." For each of the included SRMA studies, two authors scored each of the 16 questions. A third reviewer arbitrated any disputes concerning scoring. Using AMSTRA-2, the findings of this umbrella review were categorized as high, moderate, low, or critically low.

### Data analysis

The extracted data were exported to the statistical software STATA 18 to conduct statistical analysis. The overall estimates of the prevalence of preterm birth were presented using forest plots utilizing the random effects model and the Der Simonian Liard method. Furthermore, the association between different variables and preterm birth reported in each systematic review and meta-analysis study was presented using forest plots utilizing the random effects model and Hedges method. A narrative synthesis was used to present the findings of the included SRMA studies, followed by an overall meta-analysis. A meta-analysis of SRMA studies was conducted using the recommendations of Higgins et al.'s statistic (I2 = 75/100% or higher, indicating significant heterogeneity) [32]. Owing to the inclusion of a few SRMA studies, publication bias could not be assessed. To assess publication bias, at least ten studies were required.

## Results

### Search finding

**Fig. 1:**
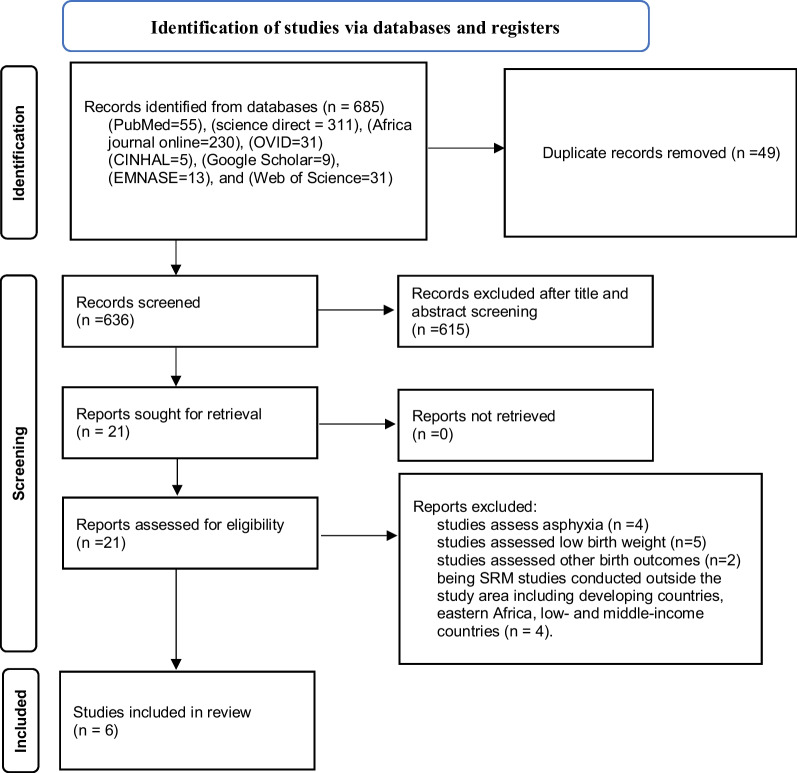
PRISMA 2020 flow diagram for searching, screening and identification of SRM studies

We were able to locate 685 articles with the aid of the major electronic medical and health databases and other relevant sources. Forty-nine articles from all identified studies were eliminated due to duplication, and 636 publications were retained for further review. Approximately 615 of these were removed after examination based on the titles and abstracts. For eligibility, 21 articles were reviewed. Approximately 15 studies were excluded because they could not show the intended result. Finally, this umbrella review comprised six systematic reviews and meta-analyses (Fig. 1).

### Characteristics of included review studies

This umbrella review aimed to examine the association between intimate partner violence (IPV) and obstetric characteristics of pregnant women with preterm birth in Ethiopia. In the included six systematic review and meta-analysis studies there are 114 primary observational studies. Of these, 89 were cross-sectional studies, 16 were case–control studies, and nine were cohort studies. The median number of studies included in each SRMA with the primary outcome was 19.5, with a minimum of 9 studies [8] to a maximum of 30 studies [13]. The median number of pregnant women for each SRMA with the primary outcome was 12,279 with a range of a minimum range of 8846 [7] to a maximum of 34,431 pregnant women [11]. This umbrella review included 75,624 pregnant women with primary outcomes. Regarding the publication status of the included SRMA studies, all SRMA studies were published in the last 5 years. Of the included SRMA studies, four examined both the prevalence and determinants of preterm birth. One study [7] only reported the prevalence of preterm births among pregnant women in Ethiopia. One SRMA study [15] reported an association between IPV and preterm births among pregnant women in Ethiopia. According to the included SRMAs studies, the reported estimate of the prevalence of preterm birth in Ethiopia ranged from 8.76% (95% CI 5.4–12.11) [7] to 13.32% (95% CI 7.99–18.66) [8]. The general characteristics of the included umbrella review of the systematic reviews and meta-analyses are shown in Table 1.

**Table 1:** Characteristics of included systematic review and meta-analysis studies in Ethiopia, 2023

Autor year	Prevalence	Sample size	Number of primary studies	Design	Registration	Quality assessment
Muchie KF et al. [12]	10.48	12,279	22	5 case–control, 2 cohort, and 15 cross-sectional studies	yes	GRADE
Sendeku FW et al. [13]	11.4	17,403	30	6 case–control, 1 cohort, and 23 cross-sectional studies	NA	NOS
Mulualem G et al. [8]	13.32	27,119	9	2 case–control, 1 cohort, and 6 cross-sectional studies	Yes	JBI
Desta M et al. [11]	13	34,431	23	5 cohort and 18 cross-sectional studies	NA	NOS
Gedefaw G et al. [7]	8.76	8846	13	13 cross-sectional studies	NA	JBI
Belay HG et al. [15]	NA	10,736	17	14 cross-sectional and 3 case–control studies	NA	NOS

*JBI* Joanna Briggs Institute checklist, *NA* Not Applicable, *NOS* Newcastle Ottawa Scale

### Quality assessment

The methodological quality of the included SRMA studies was evaluated using the AMSTAR-2 critical appraisal checklist [31]. Quality scoring was performed using 16 items, with each item scored from 0 to 2 points; the overall quality of the included studies was moderate. The quality of the included SRMAs had limitations in reporting several quality assessment items, including using PICO in the inclusion criteria, details regarding excluded studies, protocol registration, and a source of funding for the included studies (Additional file 2).

### Meta-analysis of the overall burden of preterm birth

**Fig. 2:**
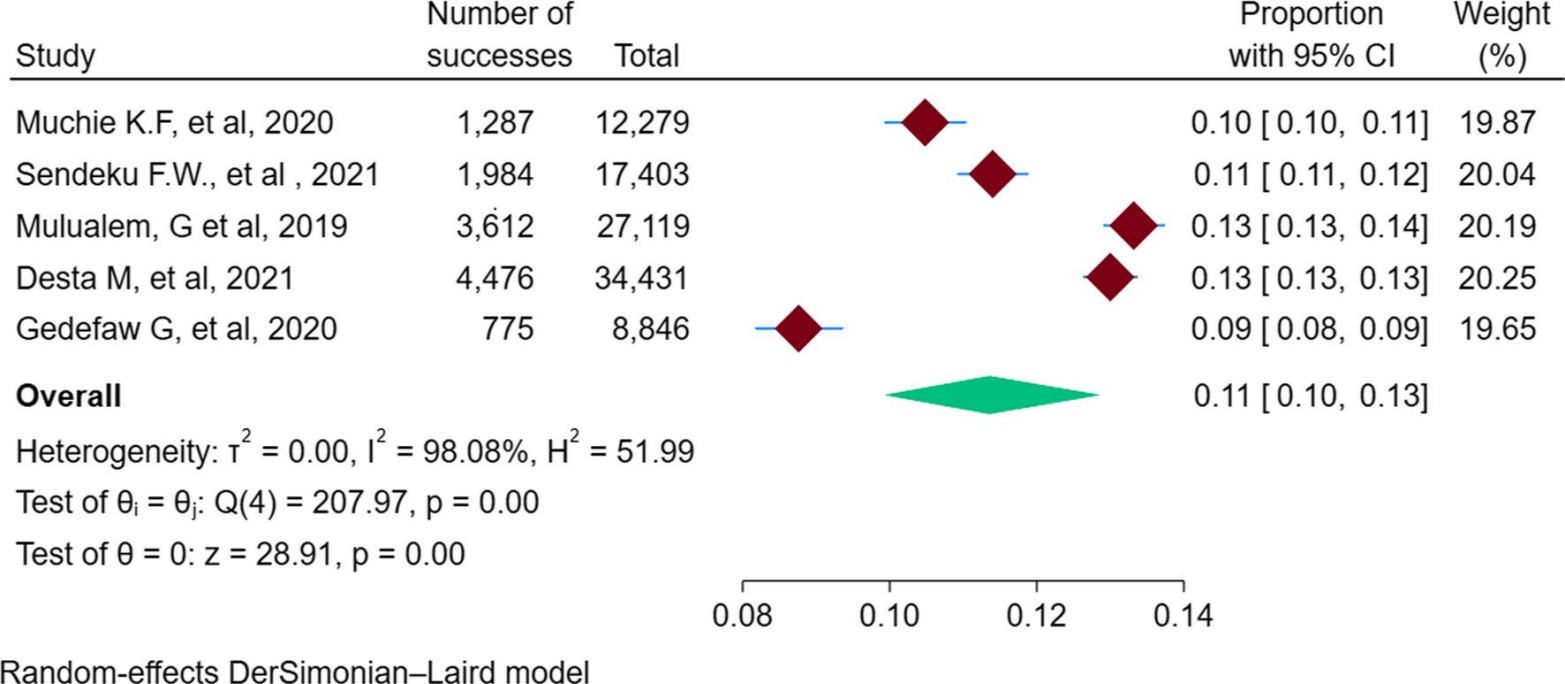
Umbrella review of systematic reviews and meta-analysis studies on the burden of preterm birth among mothers in Ethiopia, 2023

Of the six SRMA studies, five SRMA studies reported the prevalence of preterm birth among mothers and were considered for the meta-analysis of the burden of preterm birth in Ethiopia. From the umbrella review of the included SRMA studies, the overall pooled prevalence of preterm births among mothers in Ethiopia was 11% (95% CI 10–13%; I^2^ = 98.08) (Fig. 2).

### Meta-analysis on the effect of obstetrics characteristics of pregnant women on preterm birth

**Fig. 3:**
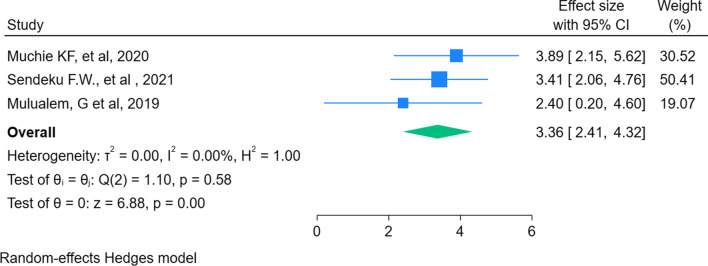
Umbrella review about the association between multiple pregnancy and preterm birth in Ethiopia, 2023

Of the six SRMA studies included, three [8, 12, 13] explored the association between multiple pregnancies and preterm births. The overall pooled estimate showed that the odds of giving preterm birth among pregnant women with multiple fetuses in utero was 3.36 times higher than pregnant women having a single fetus [POR: 3.36; 95% CI 2.41–4.32] (Fig. 3).

**Fig. 4:**
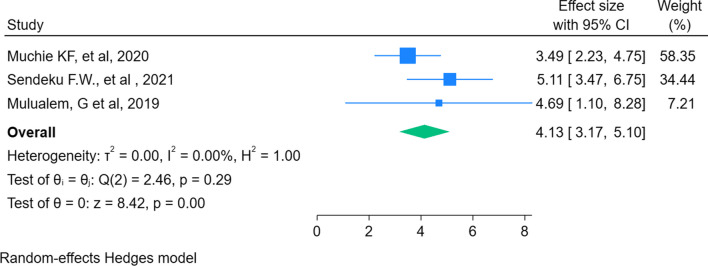
Umbrella review about the association between pregnancy-induced hypertension and preterm birth in Ethiopia, 2023

Of the included SRMA studies, three SRMA studies [8, 12, 13] examined the association between pregnancy-induced hypertensive disorder of pregnancy (PIH) and preterm birth among mothers in Ethiopia. A study done by Muchie KF, et al. showed that the odds of giving preterm birth among women having pregnancy-induced hypertension was 3.49 times higher than women without pregnancy-induced hypertension [OR = 3.49 (95% CI 2.45, 4.97]. A study done by Sendeku F.W., et al. and Mulualem, G et al. also showed that there was a statistical association between pregnancy-induced hypertension and preterm birth [OR = 5.11, 95% CI 3.73, 7.01] and [OR: 4.69; 95% CI 2.32, 9.49], respectively. The overall pooled estimate showed that the odds of giving preterm birth among pregnant women with PIH were 4.13 times higher than women without hypertensive disorders of pregnancy [POR: 4.13; 95% CI 3.17 to 5.10]. The pooled studies were homogeneous (Fig. 4).

Among the six SRMA studies, only two [12, 13] assessed the association between anemia during pregnancy and preterm rupture of membranes (PROM) with preterm birth. The overall effect size showed that anemic pregnant women were 2.76 times more likely to give preterm birth compared to non-anemic pregnant women [POR = 2.76, 95% CI 1.97–3.56]. Furthermore, the odds of giving preterm birth were 5.1 times higher among pregnant women with PROM than their counterparts [POR = 5.1, 95% CI 3.45–6.75] (Additional file 3).

The association between antepartum hemorrhage (APH) and preterm birth was studied by Muchie et al. [12]. The study showed that there is a significant association between APH and preterm birth (OR = 5.02, 95%CI 2.9–8.68). The effect of antenatal care visits during pregnancy was also explored in two SRM studies [11, 12]. A study by Muchie K.F, et al. showed that lack of antenatal care increased the risk of giving preterm birth (OR = 2.34, 95% CI 1.73–3.33). In reverse to this, a study conducted by Desta M, et al. showed that having antenatal care visits during pregnancy decreased the risk of giving preterm birth (OR = 0.39, 95% CI 0.21 0.72). Both SRM studies showed that ANC visits affected preterm birth.

### Meta-analysis on the association between intimate partner violence and preterm birth

**Fig. 5:**
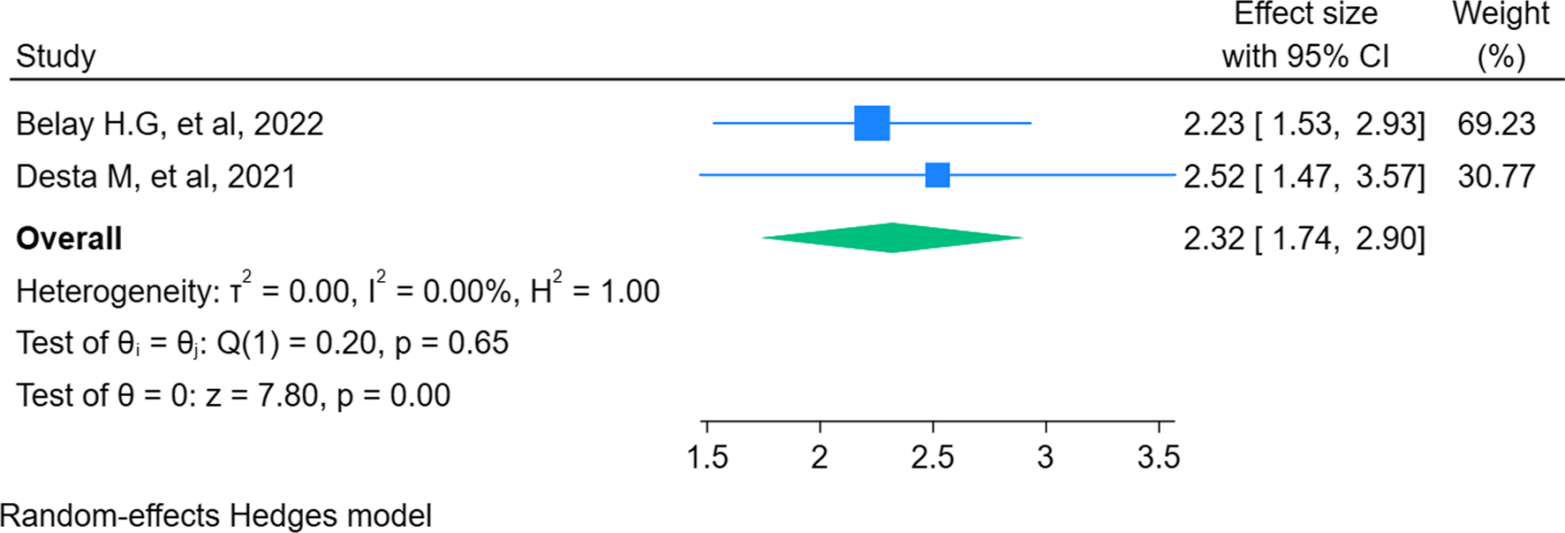
Umbrella review about the association between intimate partner violence and preterm birth in Ethiopia, 2023

Two SRMA studies [11, 15] were considered in the meta-analysis of the effect of IPV on preterm births in Ethiopia. According to a study by Belay et al., the odds of preterm birth among pregnant women who faced IPV were 2.23 times higher than in women without the condition. Similarly, a study done by Desta et al. showed that pregnant women who faced IPV were 2.52 times more likely to give preterm birth than their counterparts. The overall pooled estimate showed that pregnant women who experienced IPV were 2.32 times more likely to give preterm birth than women without IPV [POR: 2.32, 95% CI 1.74–2.90] (Fig. 5).

## Discussion

Despite extensive attempts by the Ministry of Health and non-governmental organizations, the burden of preterm births has not been reduced. This may be a result of a lack of solid and thorough data that could aid in rethinking and reevaluating the current approaches to increase the survival rate of premature neonates. Furthermore, an umbrella review of the SRMA study results must be conducted to draw more consistent conclusions, given the relevance of the topic to clinical decision-making. This umbrella review established that the overall pooled prevalence of preterm births among mothers in Ethiopia was 11% (95% CI 10–13%; I2 = 98.08). Furthermore, there is a strong relationship between intimate partner violence, multiple pregnancies, pregnancy-induced hypertension, anemia, and premature rupture of pregnancy with preterm birth.

The prevalence of preterm delivery among mothers in Ethiopia was 11% (95% CI] 10–13%; I2 = 98.08). This result is higher than those of studies conducted in Brazil (9.9% [33]), in Uganda 6.6% [34], and in sub-Saharan African countries 5.5% [33]. The overall prevalence of preterm births is also lower than studies done in Tanzania 16.6%) [34] and Nigeria 19.9%) [35]. The disparities may be due to the differences in the economic growth of the countries. Countries with higher economic growth compared to Ethiopia like Brazil may have improved facilities, and resilient health systems to combat maternal and newborn complications. Furthermore, the infrastructure may be improved which can reduce the impact of obstetric delays which have a direct impact on preterm birth. This result suggests a glaring gap between the prevention of preterm birth and its effects. The World Health Organization (WHO) emphasizes the need for a decrease in preterm birth rates because they have a significant influence on infant morbidity and mortality [36]. The Federal Ministry of Health and other stakeholders should collaborate to lessen the burden of preterm birth, its effects on the mother and fetus, the costs of hospitalization, the use of intensive care units, and care and prevention in long-term health [37]. It is also important to facilitate the implementation of preventive care protocols during antenatal care visits [21, 36, 38, 39]. Thus, to enable the implementation of necessary clinical and public health actions, the authors underlined the overall estimate revealed in this umbrella review.

We observed that there was a strong association between IPV and preterm birth. Pregnant women who experienced IPV were 2.32 times more likely to give preterm birth than women without IPV. This finding is supported by a study conducted in Zimbabwe and Australia [40, 41]. A variety of mechanisms show an association between IPV and preterm birth. Physical abuse is an IPV type that can initiate labor through mechanical factors on the abdomen and pelvis, finally resulting in preterm birth [41, 42]. Another explanation is that those who experience IPV are more likely to initiate antenatal care in the second trimester and above, which could be attributed to incorrect gestational age estimation, and will not receive preventive care during pregnancy [43]. This finding implies that there should be a preventive mechanism for IPV as well as screening of women for IPV to lessen the burden of prematurity. Furthermore, awareness creation about the impact of IPV should be strengthened.

In this study, multiple pregnancies were associated with preterm birth. The odds of preterm birth among pregnant women with multiple fetuses in utero were 3.36 times higher than those among pregnant women with a single fetus. A possible explanation could be the multifactorial nature of multiple pregnancies, which may be associated with the mechanism of labor, large placenta, cervical insufficiency, increased uterine stretch, and intrauterine infection. Multiple pregnancies cause abdominal distension. This can initiate the occurrence of arachidonic acid, and then prostaglandin synthesis, and finally preterm birth [44]. Additionally, the large placenta secretes more hormones, such as corticotrophin-releasing hormone, and fetal lung maturation products, such as surfactant protein A, which increases myometrial contractions and may cause preterm birth [45, 46]. In this study, the odds of giving preterm birth were 5.1 times higher among pregnant women with PROM than their counterparts. The possible explanation is related to the mechanical cause of labor initiation which is quite similar to the aforementioned reasons. Therefore, it is critical to implement interventions that can help prevent preterm births in multiple pregnancies [46]. Critical identification and management of multiple pregnancies and premature rupture of the membrane can be the solution to reduce preterm birth.

We found that there is a strong association between pregnancy-induced hypertension (PIH) and preterm birth. The odds of preterm birth among pregnant women with PIH were 4.13 times higher than among women without hypertensive disorders of pregnancy. This could be explained by the fact that PIH can cause stress to the fetus and mother, which can contribute to the initiation of prostaglandins, which can cause preterm labor and preterm birth [47, 48].

In this study, anemia is associated with preterm birth. Anemic pregnant women were 2.76 times more likely to give preterm birth compared to non-anemic pregnant women. This finding is supported by a meta-analysis study [49, 50]. Anemia during pregnancy is a major health problem associated with multiple adverse outcomes. Anemia may occur as a result of nutritional deficiencies. So, it is imperative to strengthen interventions that can reduce maternal malnutrition.

This umbrella review could be a solution to the lack of adequate data that hampers visibility, effective policies, and research.

## Conclusion

More than one out of ten-tenth of pregnant Ethiopian women experienced preterm birth. Intimate partner violence is significantly associated with preterm birth. Furthermore, multiple pregnancies, pregnancy-induced hypertension, anemia, and premature rupture of membranes were significant predictors of preterm birth. Therefore, policymakers should consider further instigations and implementations of policies and strategies closely related to reductions of intimate partner violence. It is also crucial to early identification and treatment of high-risk pregnancies such as multiple pregnancies, pregnancy-induced hypertension, premature rupture of membranes, and anemias to reduce the impact of preterm birth. Additionally, it is preferable to design and strengthen policies and programs that aim to improve maternal nutritional status which all contribute to a decrease in the burden of preterm birth.

### Supplementary Information

**Additional file 1. **Preferred Reporting Items for Systematic Reviews and Meta-Analyses: The PRISMA Statement Checklist.

**Additional file 2: Table S2.** Quality of assessment using AMSTAR-2

**Additional file 3: Figure S1.** Umbrella review about the association between anemia and preterm birth in Ethiopia, 2023. **Figure S2.** Umbrella review about the association between premature rupture of membrane and preterm birth in Ethiopia, 2023. **Figure S3.** Umbrella review about the association between rural residency and preterm birth in Ethiopia, 2023.

## Data Availability

The data supporting the review findings of this study are available upon submission of a reasonable request to the corresponding author.

## Abbreviations

AMSTAR: Assessment of multiple systematic reviews
MeSHs: Medical subject headings
OR: Odds ratio
PRISMA: Preferred reporting items for systematic reviews and meta-analysis
SRMA: Systematic review and meta-analysis
WHO: World Health Organization
