# Supplementary material for: Is intimate partner violence and obstetrics characteristics of pregnant women associated with preterm birth in Ethiopia? Umbrella review on preterm birth
Source: Reprod Health. 2023 Nov 17;20:168. doi: 10.1186/s12978-023-01716-7 (PMC10656915; doi:10.1186/s12978-023-01716-7)
Supplement: Supplementary file 2 — Additional file 2: Table S2. Quality of assessment using AMSTAR-2 [file 12978_2023_1716_MOESM2_ESM.docx]

**Additional file 2**

**Table 2. Quality of assessment using** AMSTAR-2

| **Authors** | **AMSTAR Items*** | | | | | | | | | | | | | | | | | |
| --- | --- | --- | --- | --- | --- | --- | --- | --- | --- | --- | --- | --- | --- | --- | --- | --- | --- | --- |
|  | **Year** | **1** | **2** | **3** | **4** | **5** | **6** | **7** | **8** | **9** | **10** | **11** | **12** | **13** | **14** | **15** | **16** | **TOTAL** |
| Muchie K.F, et al | 2020 | 0 | 1 | 1 | 1 | 2 | 2 | 0 | 1 | 1 | 2 | 2 | 2 | 2 | 2 | 2 | 2 | 22 |
| Sendeku F.W., et al | 2021 | 2 | 1 | 1 | 1 | 2 | 2 | 0 | 2 | 2 | 2 | 2 | 2 | 2 | 2 | 2 | 2 | 26 |
| Mulualem, G et al | 2019 | 0 | 1 | 0 | 1 | 2 | 2 | 0 | 2 | 1 | 2 | 2 | 2 | 2 | 2 | 2 | 2 | 22 |
| Desta M, et al | 2021 | 2 | 1 | 0 | 1 | 2 | 2 | 0 | 1 | 2 | 2 | 2 | 2 | 2 | 2 | 2 | 2 | 24 |
| Gedefaw G, et al | 2020 | 2 | 1 | 1 | 1 | 2 | 2 | 0 | 1 | 2 | 2 | 2 | 2 | 2 | 2 | 2 | 2 | 25 |
| Belay H.G, et al | 2022 | 2 | 1 | 1 | 1 | 2 | 2 | 0 | 1 | 2 | 2 | 2 | 2 | 2 | 2 | 2 | 2 | 26 |

1. *Scores for each item are between 0 and 2, where one point is awarded if the study had partial inclusion of methods that reduced bias and two points were awarded for full inclusion of methods to reduce risk of bias.
2. For full description of each individual item, access <https://amstar.ca/Amstar_Checklist.php>
