# Supplementary material for: Is intimate partner violence and obstetrics characteristics of pregnant women associated with preterm birth in Ethiopia? Umbrella review on preterm birth
Source: Reprod Health. 2023 Nov 17;20:168. doi: 10.1186/s12978-023-01716-7 (PMC10656915; doi:10.1186/s12978-023-01716-7)
Supplement: Supplementary file 3 — Additional file 3: Figure S1. Umbrella review about the association between anemia and preterm birth in Ethiopia, 2023. Figure S2. Umbrella review about the association between premature rupture of membrane and preterm birth in Ethiopia, 2023. Figure S3. Umbrella review about the association between rural residency and preterm birth in Ethiopia, 2023. [file 12978_2023_1716_MOESM3_ESM.docx]

**Additional file 2**

Additional Figure 1. Umbrella review about the association between anemia and preterm birth in Ethiopia, 2023.

Additional Figure 2. Umbrella review about the association between premature rupture of membrane and preterm birth in Ethiopia, 2023.

Additional Figure 3. Umbrella review about the association between rural residency and preterm birth in Ethiopia, 2023
